# Infliximab Reverses Suppression of Cholesterol Efflux Proteins by TNF-****α****: A Possible Mechanism for Modulation of Atherogenesis

**DOI:** 10.1155/2014/312647

**Published:** 2014-01-23

**Authors:** Iryna Voloshyna, Sangeetha Seshadri, Kamran Anwar, Michael J. Littlefield, Elise Belilos, Steven E. Carsons, Allison B. Reiss

**Affiliations:** Winthrop Research Institute and Division of Rheumatology, Allergy and Immunology, Department of Medicine, Winthrop-University Hospital, 222 Station Plaza North, Suite 502, Mineola, NY 11501, USA

## Abstract

Tumor necrosis factor- (TNF-) **α** is a proinflammatory proatherogenic cytokine. Infliximab, an anti-TNF-**α** monoclonal antibody, is effective in treating rheumatoid arthritis. However, its impact on cardiovascular burden and lipid transport is unclear. The present study investigates the effect of TNF-**α** and infliximab on reverse cholesterol transport (RCT) proteins. Uptake of modified lipoproteins by macrophages in the vasculature leads to atherogenic foam cell formation. RCT is mediated by proteins including ATP binding cassette transporters A1 (ABCA1), G1 (ABCG1), liver X receptor- (LXR-) **α**, and 27-hydroxylase. RCT counteracts lipid overload by ridding cells of excess cholesterol. THP-1 human monocytes were incubated with either TNF-**α** alone or TNF-**α** with infliximab. Expression of proteins involved in cholesterol efflux was analyzed. TNF-**α** significantly reduced both ABCA1 and LXR-**α** mRNA (to 68.5 ± 1.59%, *P* < 0.05, and 41.2 ± 0.25%, *P* < 0.01, versus control set as 100%, resp.). Infliximab nullified the TNF-**α** effect. Results were confirmed by Western blot. Infliximab abolished the increase in foam cells induced by TNF-**α**. TNF-**α** treatment significantly reduces ABCA1 and LXR-**α** expression in monocytes, thus bringing about a proatherogenic state. The anti-TNF drug infliximab, commonly used in rheumatology, restored RCT proteins. This is the first report of an atheroprotective effect of infliximab on RCT in monocytes.

## 1. Introduction

Chronic inflammatory diseases such as rheumatoid arthritis (RA) and systemic lupus erythematosus (lupus, SLE) increase cardiovascular disease (CVD) morbidity and mortality [1–3]. The antirheumatic oral disease modifying drug methotrexate is generally prescribed for first-line therapy in those with active RA [4]. Biological therapy with tumor necrosis factor (TNF)-*α* blocking agents represents an effective treatment approach for patients with RA that may be used in addition to methotrexate or as monotherapy [5]. TNF-*α* is a pleotropic and proinflammatory cytokine that has well-established effects on lipid metabolism, is expressed in atherosclerotic plaque, and has been reported to be proatherogenic [6–8]. TNF-*α* is produced primarily by activated macrophages and, to a lesser extent, by lymphocytes. Levels of TNF-*α* are elevated in the blood and synovial fluid of RA patients, and this cytokine likely plays a central role in RA pathogenesis [9]. Infliximab, a chimeric (human-mouse) monoclonal IgG1 antibody against TNF-*α*, binds with high affinity to both soluble and membrane-bound TNF-*α*, thus neutralizing its biological activity [10].

Although methotrexate has been shown to reduce CVD risk in RA, the effects of anti-TNF therapy on CVD comorbidity are not completely known. Recent studies have shown that treatment with anti-TNF agents modifies cardiovascular burden in patients with rheumatoid arthritis via an increase in HDL and total cholesterol [11]. Triglycerides may also increase, but the long-term effects of TNF-*α* blockade on lipid pattern are still unclear [12].

In this report, we demonstrate the effect of TNF-*α* and infliximab on the reverse cholesterol transport (RCT) proteins. Uptake of modified lipoproteins by macrophages in the vasculature leads to cholesterol overload and formation of atherogenic foam cells [13]. RCT is a process of cellular cholesterol efflux mediated by specific proteins including ATP binding cassette transporter A1 and G1 (ABCA1 and ABCG1) and liver X receptor *α* (LXR-*α*) [14–16]. These two proteins counteract foam cell formation by ridding cells of excess cholesterol. The cholesterol 27-hydroxylase represents an intracellular cholesterol elimination pathway. It converts extrahepatic cholesterol into 27-hydroxycholesterol and 3*β*-hydroxy-5-cholestenoic acid, oxysterol metabolites that are polar and therefore more readily excreted from cells than cholesterol. This study demonstrates that TNF-*α* and infliximab exhibit pro- and antiatherogenic effects, respectively, via effects on expression of genes involved in RCT.

## 2. Materials and Methods

### 2.1. Cells and Reagents

THP-1 monocytes were obtained from American Type Culture Collection (Manassas, VA).

Phorbol 12-myristate 13-acetate (PMA) and Oil red O were purchased from Sigma-Aldrich (St. Louis, MO).

Trizol reagent was purchased from Invitrogen (Grand Island, NY).

All reagents for reverse transcription-polymerase chain reaction (RT-PCR) were purchased from Applied Biosystems (Chicago, IL). FastStart SYBR Green Master mix for the quantitative real-time polymerase chain reaction (QRT-PCR) was obtained from Roche Applied Science (Indianapolis, IN). Primers used in amplification reactions were generated by Sigma-Genosys (The Woodlands, TX).

The BCA Protein Assay Kit and all reagents for Western blot protein detection were purchased from Thermo Scientific Pierce Biotechnology Inc. (Rockford, IL).

Anti-cholesterol 27-hydroxylase antibody is an affinity-purified rabbit polyclonal antipeptide antibody raised against residues 15–28 of the cholesterol 27-hydroxylase protein [17].

Rabbit anti-human ABCA1 (sc-20794) and LXR-*α* (sc-20) antibody were obtained from Santa Cruz Biotechnology (Santa Cruz, CA). Rabbit anti-human ABCG1 (ab-36969) and *β*-actin (ab-6276) antibody were purchased from Abcam Inc. (Cambridge, MA). Donkey anti-rabbit horseradish peroxidase (HRP) linked antibody and sheep anti-mouse IgG-HRP conjugated antibody, used as a secondary antibody, were purchased from GE Healthcare Biosciences (Piscataway, NJ).

### 2.2. Cell Culture and Experimental Conditions

The THP-1 human monocytes were cultured in suspension in RPMI 1640 medium with 10% fetal calf serum (FCS) at 37°C in a 5% CO_2_ atmosphere. Studies were performed at a density of 1 × 10^6^ cells/mL. THP-1 cells were incubated (18 hr, 37°C, 5% CO_2_, and *n* = 3 per condition) under the following conditions: (1) untreated control (media alone), (2) interferon (IFN)-*γ* (500 U/mL), (3) TNF-*α* (100 U/mL), (4) TNF-*α* + infliximab (5 *μ*g/mL), and (5) infliximab (5 *μ*g/mL). After incubation, media were aspirated, and cellular RNA and protein were isolated.

To facilitate differentiation into macrophages, THP-1 monocytes were treated with 100 nM PMA for 48 h. When differentiated phenotype was achieved, the PMA-containing medium was removed, replaced with complete RPMI 1640, supplemented with 10% FCS, and used for the oxidized (ox)LDL accumulation assay and foam cell transformation analysis.

### 2.3. RNA Extraction and QRT-PCR

Total RNA was extracted using 1 mL Trizol reagent per 10^6^ cells and dissolved in nuclease-free water. The quantity of total RNA from each condition was measured by absorption at 260 and 280 nm wavelengths by ultraviolet spectrophotometry (Hitachi U2010 spectrophotometer). For each assay 1 *μ*g of RNA was reverse transcribed using Murine Leukemia Virus reverse transcriptase primed with oligo dT in an Eppendorf Mastercycler-personal PCR thermocycler (Eppendorf, Hamburg, Germany). Equal amounts of cDNA were taken from each RT reaction mixture for PCR amplification using the following specific primers:

 ABCA1 forward 5′-GAAGTACATCAGAACATGGGC-3′
 ABCA1 reverse 5′-GATCAAAGCCATGGCTGTAG-3′
 ABCG1 forward 5′-CAGGAAGATTAGACACTGTGG-3′
 ABCG1 reverse 5′-GAAAGGGGAATGGAGAGAAG-3′;
 27-hydroxylase forward 5′-AAGCGATACCTGGATGGTTG-3′
 27-hydroxylase reverse 5′-TGTTGGATGTCGTGTCCACT-3′
 LXR*α* forward 5′-GGGGCCAGCCCCCAAAATGCTG-3′
 LXR*α* reverse 5′-GCATCCGTGGGAACATCAGTCG-3′.

QRT-PCR analysis was performed using the FastStart SYBR Green Reagent Kit according to the manufacturers' instructions on the Roche Light Cycler 480 (Roche Applied Science, Indianapolis, IN). Each reaction was done in triplicate. The amounts of PCR products were estimated using Roche Applied Science software, provided by the manufacturer. Fluorescence emission spectra were monitored and analyzed. PCR products were measured by the threshold cycles (*C*_*T*_), at which specific fluorescence becomes detectable. The *C*_*T*_ value for each gene was normalized by that for glyceraldehydes-3-phosphate dehydrogenase (GAPDH), and the relative expression level was calculated as the mean value of the untreated THP-1 as 1. Nontemplate controls were included for each primer pair to check for significant levels of any contaminants. A melting-curve analysis was performed to assess the specificity of the amplified PCR products.

### 2.4. Protein Extraction and Western Blot Analysis

Total cell lysates were prepared for Western blotting using RIPA lysis buffer (98% PBS, 1% Igepal CA-630, 0.5% sodium deoxycholate, and 0.1% sodium dodecyl sulfate (SDS)), containing protease inhibitor cocktail (Sigma) (10 *μ*L per 100 *μ*L of RIPA buffer). Cell pellets from each condition were incubated on ice for 35 min with vortexing every 5 min. Supernatants were collected after centrifuging at 10,000 g at 4°C for 10 min using an Eppendorf 5415C centrifuge. The quantity of protein in each supernatant was measured by absorption at 560 nm using a Hitachi U2010 spectrophotometer.

Protein samples (20 *μ*g/lane) were boiled for 5 minutes, fractionated on 8% SDS-PAGE, and transferred onto a PDVF membrane (Bio-Rad, Hercules, CA). The membrane was blocked for an hour at room temperature in blocking solution (5% nonfat dry milk (Bio-Rad) in 1X Tris-buffered saline/1% Tween 20 [TTBS]) and then immersed in different dilutions of primary antibody for protein detection. To detect and quantify expression of 27-hydroxylase, a 1 : 300 dilution of primary antibody in blocking solution was used overnight at 4°C. 1 : 500 dilutions of primary antibody were used for detection of ABCA1, ABCG1, and LXR-*α*.

The following day, the membrane was washed and then incubated in a 1 : 5000 dilution of ECL HRP-linked donkey anti-rabbit antibody in blocking solution. As a control, on the same transferred membrane, beta-actin was detected using mouse anti-beta-actin primary antibody at 1 : 1000 dilution and ECL sheep anti-mouse-IgG HRP-linked species-specific whole antibody, diluted 1 : 5000 (Amersham Biosciences, Piscataway, NJ) and subjected to all other similar steps as above.

The immunoreactive proteins were detected using Pierce ECL Western Blot substrate system and film development in SRX-101A (Konica Minolta Holdings, Inc., Tokyo, Japan). Stripping and reprobing of the membranes were performed according to the manufacturer's protocol (Thermo Scientific, Rockford, IL).

Band intensities for Western blot protein samples were quantified using Kodak Digital Science 1D, version 2.0.3, after imaging with Kodak Digital Science Electrophoresis Documentation and Analysis System 120.

### 2.5. Cholesterol Efflux Analysis

Cholesterol efflux to HDL was analyzed when THP-1 macrophages were exposed to human HDL (Intracel, Frederick, MD) in RPMI for 6 h (100 *μ*g/mL) subjected to all conditions described above. After incubation, both extracellular (in cell growth medium) and intracellular (in cells) total (TC) and free (FC) cholesterol were analyzed. The Amplex Red Cholesterol Assay Kit (Molecular Probes, Eugene, OR) was used according to the manufacturer's protocol. Cholesterol esters (CE) were calculated as the difference between TC and FC. The HDL-mediated net cholesterol efflux was calculated by subtraction of the cholesterol mass of the medium from that of the cells. CE/FC ratio was calculated.

### 2.6. Foam Cell Formation Assay

THP-1 differentiated macrophages were cholesterol-loaded with 50 *μ*g/mL acetylated LDL or 50 *μ*g/mL oxLDL (Intracel, Frederick, MD) for 24 h and subjected to conditions described above for another 24 h in the presence/absence of modified LDL. For Oil Red O staining, cells were fixed in 4% paraformaldehyde and then washed and stained with 0.2% Oil Red O (Sigma) for 30 min. After the PBS wash, cell nuclei were stained with hematoxylin (Sigma) for 5 min. After a final wash with PBS, coverslips were mounted on slides using Permount solution (Sigma).

Foam cells, recognized as macrophages stained with Oil Red O, were visualized via light microscopy (Axiovert 25; Carl Zeiss, Gottingen, Germany) with 40x magnification and photographed using a DC 290 Zoom digital camera (Eastman Kodak, Rochester, NY). The number of foam cells formed in each condition was calculated in triplicate manually and presented as percentage of total cells.

### 2.7. Internalization of OxLDL

THP-1 differentiated macrophages were incubated with 50 *μ*g/mL oxLDL (Intracel, Frederick, MD) for 24 h and subjected to conditions described above in the presence of 5 *μ*g/mL 1,1′-dioctadecyl-3,3,3′,3′-tetramethylin docarbocyaninet (Dil)-oxLDL (Intracel, Frederick, MD) for another 3 h. Following incubation, media were aspirated, and slides were washed with PBS and fixed in 4% paraformaldehyde in water for 15 min. After washing, accumulation of Dil-oxLDL in cells was determined by fluorescent intensity incorporated within cells using a Nikon A1 microscopy unit at 40x magnification. Cells were photographed with a DS-Ri1 digital camera. Fluorescent intensity was quantified from at least 3 random fields (1024 × 1024 pixels) per slide, from 3 slides per experimental condition, and graphed.

### 2.8. Statistical Analysis

Statistical analysis was performed using Graphpad Prism, version 5.01. All data were analyzed by one-way analysis of variance, and pairwise multiple comparisons were made between control and treatment conditions using Bonferroni correction. *P *values less than 0.05 were considered significant.

## 3. Results and Discussion

In all experiments, treated THP-1 monocytes were compared with untreated control cells. The mRNA and protein expression of untreated controls were set at 100%.

**Figure 1:**
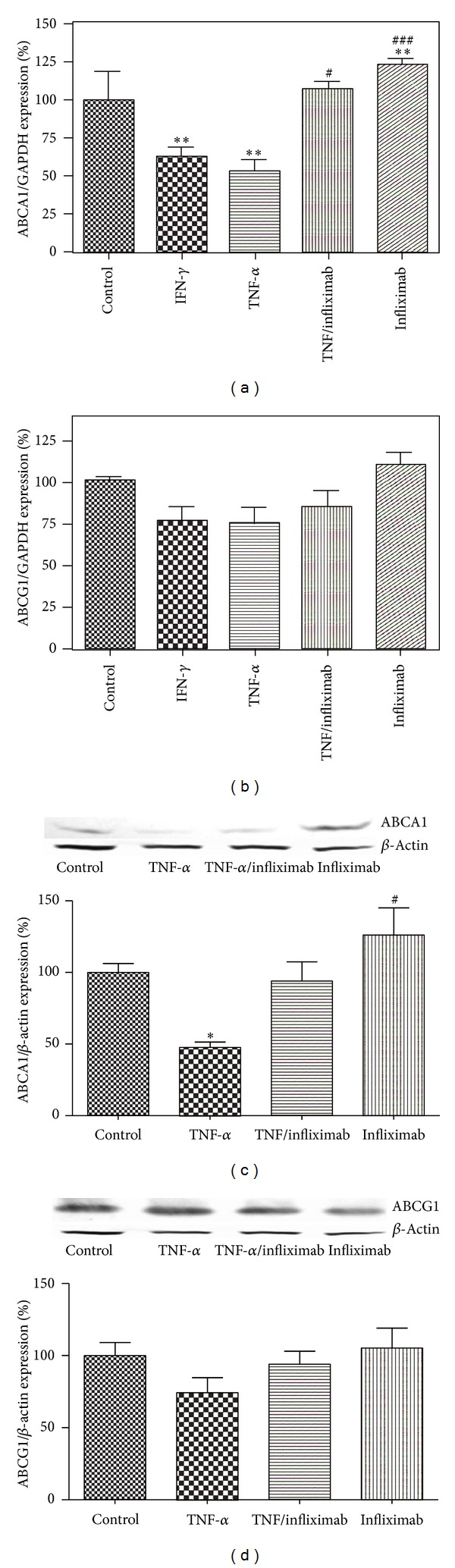
ABCA1 message (a) and protein (c) levels in THP-1 monocytes decrease with exposure to TNF-*α* and this effect is reversed by infliximab. mRNA level (b) and protein expression (d) of ABCG1 did not change in the presence of TNF-*α* or infliximab. THP-1 monocytes were incubated under the following 5 conditions: (1) RPMI alone (Control), (2) IFN-*γ* (500 U/mL), (3) TNF-*α* (100 U/mL), (4) TNF-*α* (100 U/mL) + infliximab (5 *μ*g/mL), and (5) infliximab (5 *μ*g/mL). Quantitative analysis for changes in ABCA1 and ABCG1 mRNA level was performed using RT-PCR with GAPDH message as an internal standard. Protein expression was analyzed by Western blot and band intensities were calculated using *β*-actin as a loading control. All results are presented as means ± SEM of three independent experiments. *P < 0.05; **P < 0.01; ***P < 0.001 versus control; ^#^ *P* < 0.05; ^###^ *P* < 0.001 versus TNF-*α* treated cells.

Our study demonstrates beneficial effect of infliximab on cholesterol efflux disrupted by exposure of THP-1 monocytes to TNF-*α*. As previously reported [17, 18], IFN-*γ* reduced ABCA1 gene and protein expression to 63.2 ± 5.2% and 67.5 ± 5.2% (not shown) versus control (*P* < 0.01, *n* = 3), respectively (Figure 1(a)). Similarly, TNF-*α* treatment significantly diminished ABCA1 mRNA level to 53.5 ± 1.6% (*P* < 0.01, *n* = 3) and protein to 50.7 ± 6.6% (*P* < 0.05, *n* = 3) versus control (Figures 1(a) and 1(c)). However, mRNA and protein levels of ABCG1 did not change significantly upon exposure to IFN-*γ* or TNF-*α* (NS, *n* = 3) (Figures 1(b) and 1(d)). The presence of infliximab in TNF-*α* treated cells nullified the TNF-*α* effect by increasing ABCA1 to the level of control and to double the expression versus TNF-*α* alone (*P* < 0.001, *n* = 3). Exposure of THP-1 monocytes to infliximab alone increased the level of ABCA1 to 128.8 ± 8.3% versus control (*P* < 0.01, *n* = 3) as well and did not affect expression of ABCG1 (Figure 1).

**Figure 2:**
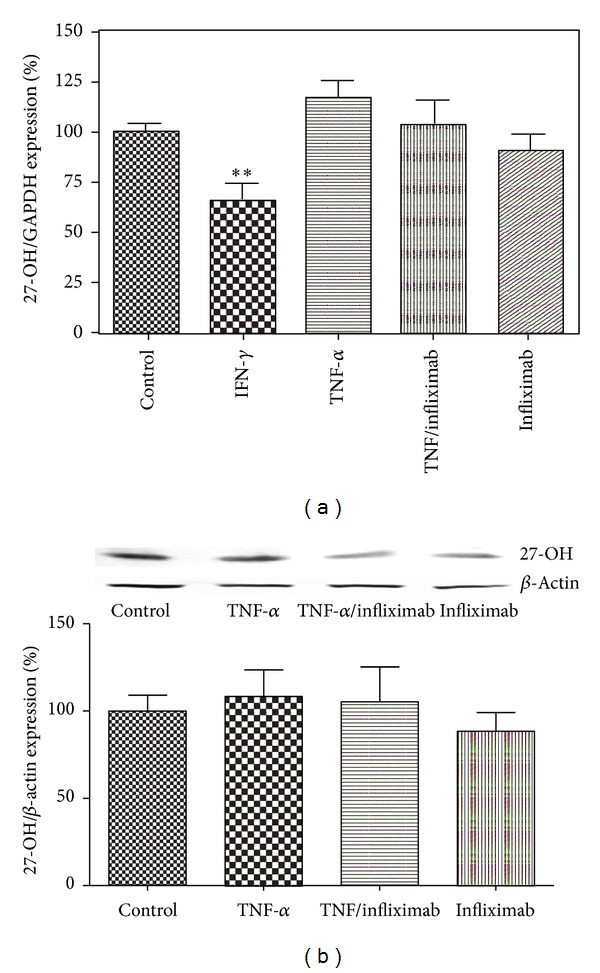
The effect of TNF-*α* and infliximab on 27-hydroxylase (27-OH) message (a) and protein (b) levels in THP-1 monocytes. THP-1 monocytes were incubated under the following 5 conditions: (1) RPMI alone (Control), (2) IFN-*γ* (500 U/mL), (3) TNF-*α* (100 U/mL), (4) TNF-*α* (100 U/mL) + infliximab (5 *μ*g/mL), and (5) infliximab (5 *μ*g/mL). Quantitative analysis for changes in ABCA1 mRNA level was performed using RT-PCR with GAPDH message as an internal standard. Protein expression analysis was performed using Western blot and band intensities were calculated using *β*-actin as a loading control. All results are presented as means ± SEM of three independent experiments. **P < 0.01; versus control cells.

27-hydroxylase expression was affected by IFN-*γ*, reducing mRNA level to 60.5 ± 6.1% versus control. In contrast, TNF-*α* or infliximab did not significantly change mRNA and protein expression of 27-hydroxylase (Figure 2).

**Figure 3:**
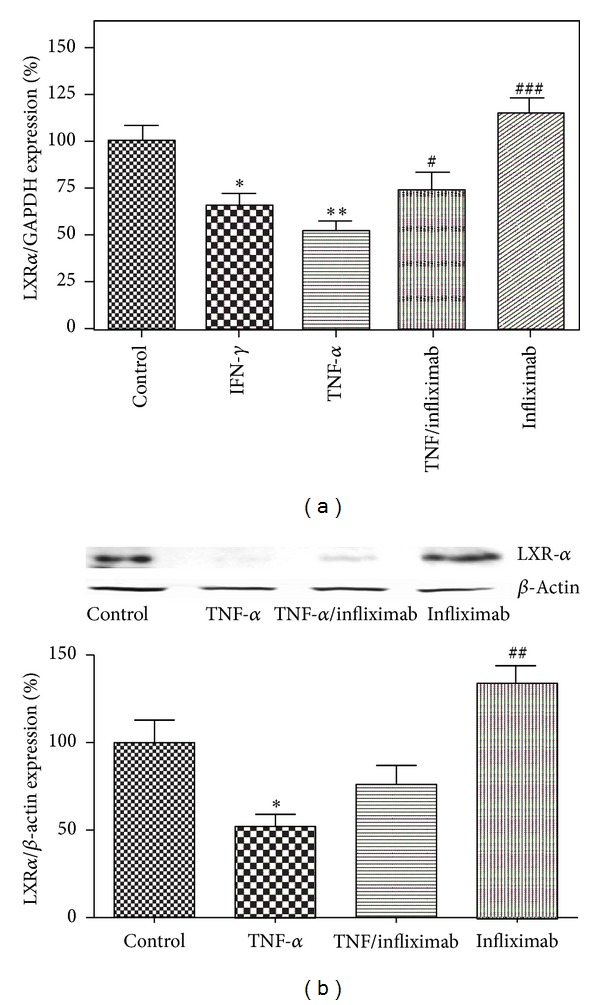
LXR-*α* message (a) and protein level (b) in THP-1 monocytes decrease with exposure to TNF-*α* and this effect is reversed by infliximab. THP-1 monocytes were incubated under the following 5 conditions: (1) RPMI alone (Control), (2) IFN-*γ* (500 U/mL), (3) TNF-*α* (100 U/mL), (4) TNF-*α* (100 U/mL) + infliximab (5 *μ*g/mL), and (5) infliximab (5 *μ*g/mL). Quantitative analysis for changes in TNF-*α* expression was performed using RT-PCR with GAPDH message as an internal standard. All results are presented as means ± SEM of three independent experiments. *P < 0.05; **P < 0.01; versus control; ^#^ *P* < 0.05; ^##^ *P* < 0.01; ^##^ *P* < 0.001 versus TNF-*α* treated cells.

Alterations in LXR-*α* expression followed the same pattern as ABCA1. IFN-*γ* treatment declined expression of LXR-*α* by 34.6 ± 3.2% versus control (Figure 3(a)), TNF-*α* downregulated mRNA and protein level of LXR-*α* to 41.2 ± 2.5% and 53.2 ± 8.7% (*P* < 0.01, *n* = 3), respectively (Figure 3). Addition of infliximab to cells treated with TNF-*α* augmented LXR-*α* expression to 124.7 ± 1.7% for mRNA and 137.2 ± 7.6% for protein (*P* < 0.05, *n* = 3), respectively, versus control and doubled LXR-*α* levels versus cells exposed to TNF-*α* alone (*P* < 0.001, *n* = 3) (Figure 3).

The infliximab concentration used in this study is within the therapeutic range for RA and is close to the average trough level of 3.3 *μ*g/mL [19, 20]. Our finding that TNF-*α* decreases ABCA1 and LXR-*α* is consistent with previous reports in macrophages and other cell types [21–24]. However, here we present the first report of the TNF-*α* effect in THP-1 monocytes and its specific augmentation with infliximab.

**Figure 4:**
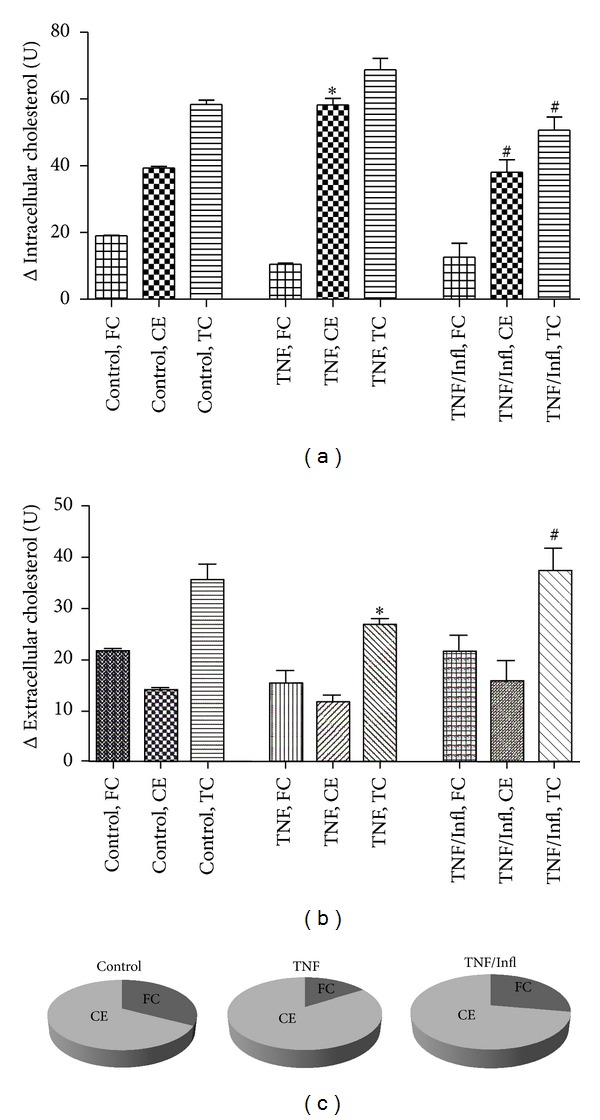
TNF-*α* represses net cholesterol efflux in THP-1 macrophages. Net cholesterol efflux to HDL [100 *μ*g/mL] is reduced in the presence of TNF-*α*, increasing intracellular total cholesterol mass and amount of cholesterol esters (CE) in THP-1 macrophages (a). Simultaneously extracellular cholesterol is reduced upon exposure to TNF-*α* (b). TNF-*α* exposed cells display an increase in the intracellular ratio of CE/FC versus control cells (c). The presence of infliximab abolishes the effect of TNF-*α* on cholesterol efflux, decreasing the CE/FC ratio and maintaining efflux similar to control cells. Values are mean of three independent experiments. *P < 0.05, versus control cells; ^#^ *P* < 0.05, versus TNF-*α* treated cells.

Next we analyzed the effect of TNF-*α* and infliximab on cholesterol efflux to the extracellular acceptor—HDL. Cholesterol mass in the media was quantitated fluorometrically using the Amplex Red Cholesterol Assay Kit. We observed that TNF-*α* augmented the ability of THP-1 macrophages to remove cholesterol from cells to the medium (Figure 4). Thus, in the cells exposed to TNF-*α*, HDL-mediated efflux was decreased and this resulted in accumulation of intracellular cholesterol (mainly CE), decreasing total cholesterol mass in the media versus control cells (*n* = 3, *P* < 0.05) (Figures 4(a) and 4(b)). Moreover, TNF-*α* exposed cells display an increase in the intracellular ratio of CE/FC versus control cells (Figure 4(c)). Cholesterol esters (CE) are much less polar than free cholesterol (FC) and are major constituents of the fatty lesions in atherosclerotic plaques. The presence of infliximab abolished the effect of TNF-*α* on cholesterol efflux, decreasing the CE/FC ratio and maintaining efflux similar to control cells. Interestingly, the level of FC did not differ significantly in all treated groups, corresponding to earlier noted lack of significant changes in ABCG1 level upon TNF ± infliximab exposure. ABCG1-mediated efflux is known to be the main pathway for FC elimination [25].

**Figure 5:**
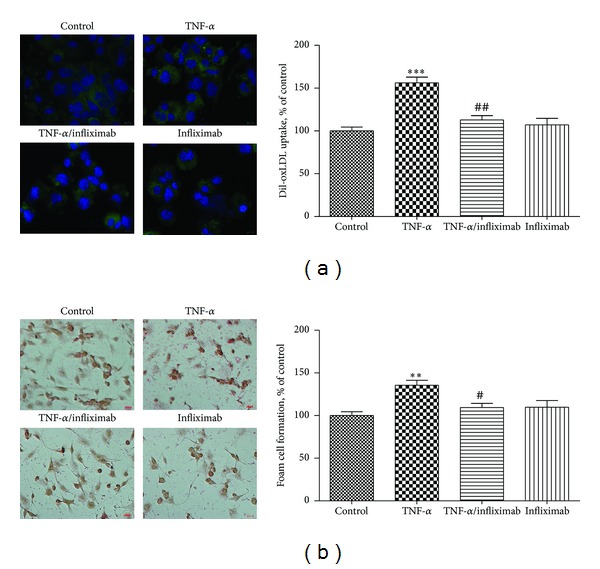
Infliximab nullifies augmented oxLDL uptake and accumulation in THP-1 macrophages induced by TNF-*α*. (a) THP-1 differentiated macrophages were incubated with 50 *μ*g/mL oxLDL for 18 h, followed by an additional 3 h in the presence of 5 *μ*g/mL Dil-oxLDL and RPMI alone (Control), TNF-*α* (100 U/mL), TNF-*α* (100 U/mL) + infliximab (5 *μ*g/mL), and infliximab (5 *μ*g/mL). Dil-oxLDL accumulation was measured by fluorescent intensity with a Nikon A1 microscopy unit with 40x magnification and photographed with a DS-Ri1 digital camera. Fluorescent intensity was quantified from at least 3 random fields (1024 × 1024 pixels) per slide, from 3 slides per experimental condition, and graphed. (b) THP-1 differentiated macrophages were incubated with 50 *μ*g/mL oxLDL for 18 h, followed by an additional 18 h under the conditions detailed in part (a) above. Foam cell formation (FCF) was calculated as a percentage of Oil-Red-O stained cells. All results are presented as a percent of control cells with control set at 100% and expressed as mean ± SEM of five independent experiments. **P < 0.01; ***P < 0.001 versus control cells; ^#^ *P* < 0.05; ^##^ *P* < 0.01 versus TNF-*α* treated cells.

As a physiologic correlate to our expression studies, we show a substantial dampening effect of infliximab on foam cell formation and modified lipid uptake in TNF-*α*-treated macrophages (Figure 5). Although RCT proteins do not directly impact lipid uptake, their enhancement improves overall lipid balance in the cells by providing a path for egress. Thus, incubation of macrophages with TNF-*α* increased accumulation of DiI-oxLDL to 156.6 ± 7.8% (*P* < 0.001, *n* = 9) versus control cells and this effect was abrogated by the presence of infliximab to the level of control (Figure 5(a)). Correspondingly, the presence of TNF-*α* amplified transformation of THP-1 macrophages into foam cell by 34.8% as compared to control cells (*P* < 0.0, *n* = 5) (Figure 5(b)). Infliximab kept the foam cell formation at the level of control cells (Figure 5). Similar results were obtained when acetylated LDL was used for loading of THP-1 macrophages (data not shown).

One of the most compelling clinical challenges in the management of RA is the high incidence of atherosclerotic CVD [1, 2]. At this time, clinicians cannot predict which of their patients are most likely to suffer myocardial infarction, and treatment options involve strategies to minimize inflammation and reduce traditional risk factors [23, 24]. New biologicals may offer further protection. In this cell culture study, we begin to address the issue of cardiovascular effects of anti-TNF agents. Critical to the atherosclerotic process is dysfunction of the RCT mechanism which clears excess cholesterol from the artery. In cultured THP-1 monocytes, exposure to TNF-*α* diminishes message level of both ABCA1 and LXR. Infliximab reverses the effects of TNF-*α* by increasing both ABCA1 and LXR-*α* gene expression, thereby restoring cholesterol balance. Since LXR-*α* is the receptor through which ABCA1 is regulated, ABCA1 may be increasing via LXR signaling [14]. This study enhances our understanding of the interplay between autoimmunity and atherogenesis in a way that leads us to appreciate the potential for TNF-*α* inhibiting drugs to prevent CVD in high risk patients with the immunologically mediated disease RA.

Recently, a number of approaches to restoration of cholesterol balance in autoimmune rheumatic diseases have been explored. Promising possibilities include activation of specific adenosine pathways through either synthetic agonists or nutraceuticals such as the polyphenolic antioxidant resveratrol [26, 27]. Both adenosine receptor agonists and resveratrol have been found to increase RCT proteins and resveratrol was shown to inhibit TNF-*α* effects on RA synovial fibroblasts [28]. The combination of infliximab with these other agents may be an area of further study in the pursuit of better ways to reduce CVD risk in RA.

## 4. Conclusions

TNF-*α* treatment of monocytes dramatically and significantly reduces ABCA1 and LXR gene expression leading to a proatherogenic effect. Infliximab, an anti-TNF drug commonly used in rheumatology, reversed the effects of TNF-*α* by increasing both ABCA1 and LXR gene expression, thereby potentially ameliorating RCT. This is the first report indicating an atheroprotective effect of infliximab in monocytes. Future studies are needed to evaluate infliximab effects on RCT genes in vivo.
